# Supplementary material for: A Novel Photoluminescent Ag/Cu Cluster Exhibits a Chromic Photoluminescence Response towards Volatile Organic Vapors
Source: Molecules. 2023 Jan 27;28(3):1257. doi: 10.3390/molecules28031257 (PMC9921385; doi:10.3390/molecules28031257)
Supplement: Supplementary file 1 [file molecules-28-01257-s001.zip › molecules-2151837-supplementary.pdf]

Supporting materials

A Novel Ag/Cu Cluster Exhibits a Chromic Photoluminescence Response towards Volatile Organic Vapors

Wei Yang, Shengnan Hu, Yuwei Wang, Sisi Yan, Xiang-Qian Cao, Hong-Xi Li, David James Young, and Zhi-gang Ren*

Contents

Figure S1. IR spectra of **1** and **1a**.

Figure S2. Experimental and simulated isotopic patterns in the positive ion ESI-MS spectra of **1** in MeOH.

Figure S3. TGA curve of **1** in a N_2 stream.

Figure S4. Emission wavelengths of **1a** over four cycles after exposure to EtOH vapor and vacuum heating.

Figure S5. PXRD patterns of as synthesized **1a**, **1a** after four EtOH exposure/elimination cycles and being left in air for another 1 month.

Figure S6. Emission spectra of **1a** in air (**1a**) and its powder after immersed in water for 20 hours (**1a-water**).

Table S1. Selected bond lengths and angles for **1**·$2CH_2Cl_2$.

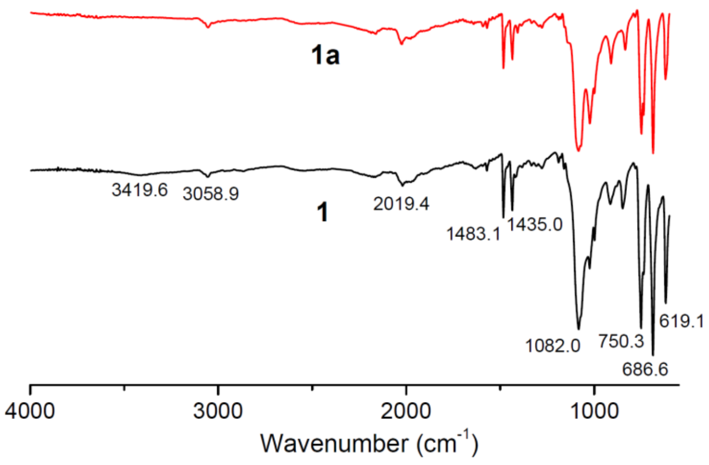

Figure S1. IR spectra of **1** and **1a**.

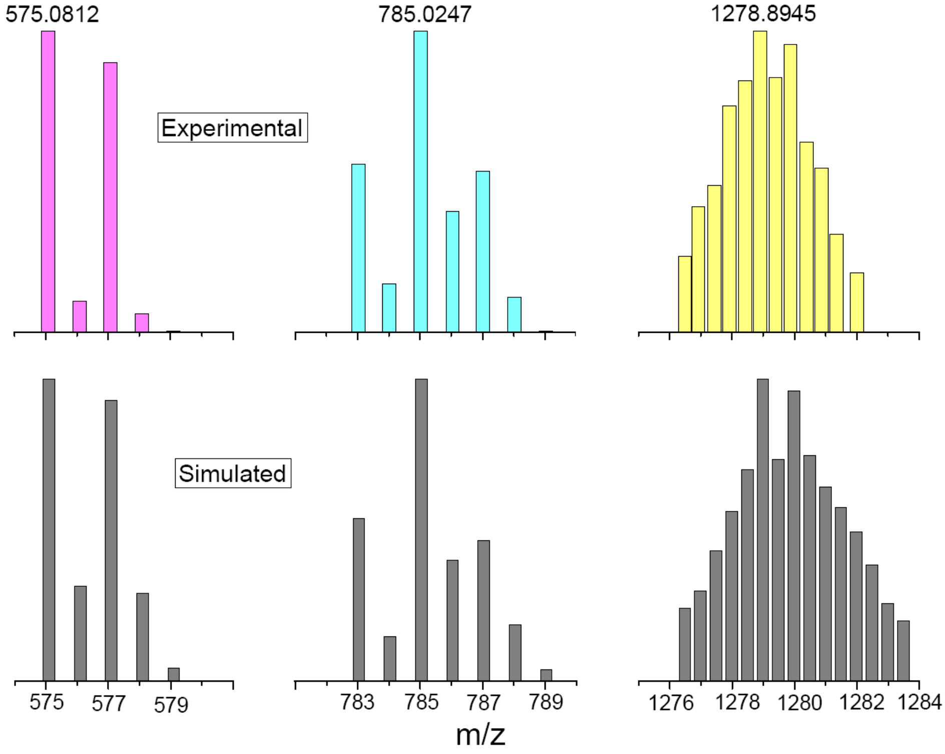

Figure S2. Experimental and simulated isotopic patterns in the positive ion ESI-MS spectra of **1** in MeOH.

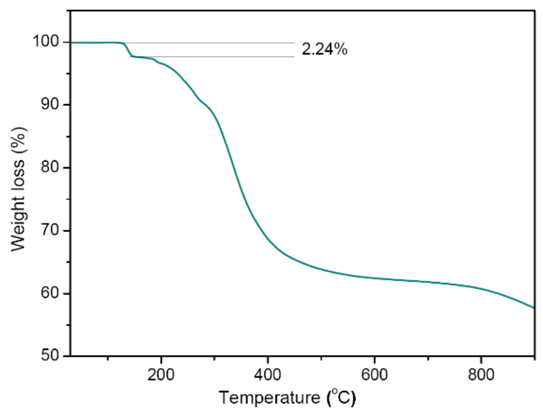

Figure S3. TGA curve of **1** under a N_2 stream.

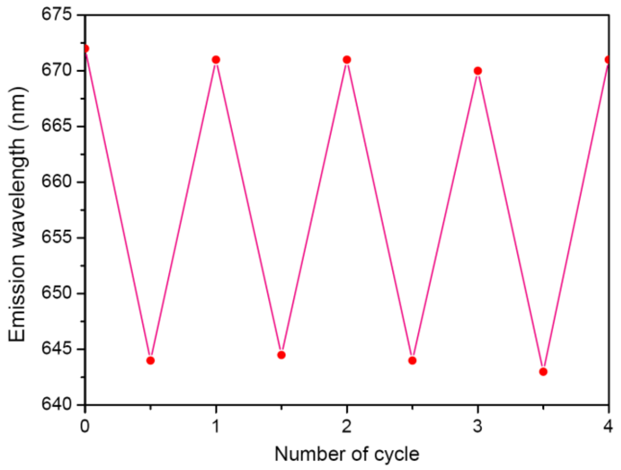

Figure S4. Emission wavelengths of **1a** over four cycles after exposure to EtOH vapor and vacuum heating.

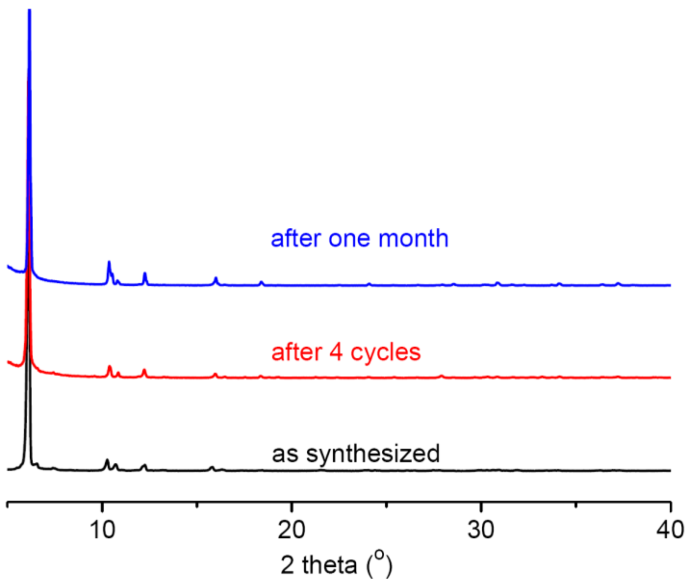

Figure S5. PXRD patterns of as synthesized **1a** (black), **1a** after four EtOH exposure/elimination cycles (red) and being left in air for another 1 month (blue).

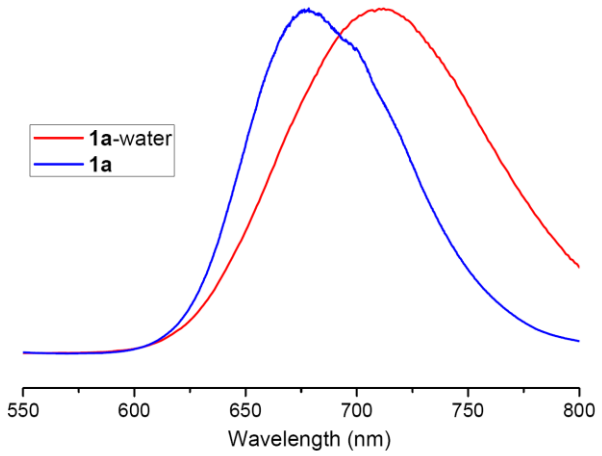

Figure S6. Emission spectra of **1a** in air (**1a**) and its powder after immersed in water for 20 hours (**1a-water**) under 400 nm excitation.

Table S1. Selected bond lengths (Å) and angles (°) for **1**·$2CH_2Cl_2$.

Cu(2)-N(1)	2.299(9)	Ag(1)-Cu(1)	2.7505(19)	Ag(8)-Cu(3)	2.778(2)	Ag(1)-Ag(10)	2.9792(14)
Cu(3)-N(2)	2.293(9)	Ag(1)-Cu(2)	2.824(2)	Ag(8)-Cu(4)	2.8437(19)	Ag(1)-Ag(5)	3.1571(16)
Cu(5)-N(3)	2.224(8)	Ag(2)-Cu(3)	2.701(2)	Ag(8)-Cu(5)	2.9147(18)	Ag(2)-Ag(7)	2.9420(14)
Cu(6)-N(4)	2.267(9)	Ag(2)-Cu(4)	2.749(2)	Ag(9)-Cu(3)	3.180(2)	Ag(2)-Ag(8)	3.2486(15)
Cu(1)-C(59)	1.929(13)	Ag(3)-Cu(5)	2.8998(19)	Ag(9)-Cu(5)	2.9111(19)	Ag(3)-Ag(7)	3.1801(15)
Cu(1)-C(67)	1.954(12)	Ag(3)-Cu(4)	2.9137(19)	Ag(9)-Cu(6)	2.8395(19)	Ag(3)-Ag(8)	2.9605(13)
Cu(2)-C(75)	1.871(13)	Ag(4)-Cu(1)	2.835(2)	Ag(10)-Cu(1)	2.8845(19)	Ag(4)-Ag(5)	2.9180(13)
Cu(2)-C(83)	1.889(13)	Ag(4)-Cu(6)	2.814(2)	Ag(10)-Cu(2)	2.8582(18)	Ag(5)-Ag(6)	3.3104(18)
Cu(3)-C(91)	1.915(13)	Ag(5)-Cu(1)	2.724(2)	Ag(10)-Cu(6)	2.887(2)	Ag(5)-Ag(10)	2.8292(15)
Cu(3)-C(99)	1.909(12)	Ag(5)-Cu(2)	2.9835(19)	Ag(1)-P(1)	2.402(3)	Ag(6)-Ag(7)	3.1007(18)
Cu(4)-C(107)	1.916(13)	Ag(6)-Cu(3)	3.121(2)	Ag(2)-P(2)	2.389(3)	Ag(6)-Ag(9)	2.9077(15)
Cu(4)-C(115)	1.960(13)	Ag(6)-Cu(5)	2.878(2)	Ag(3)-P(3)	2.379(3)	Ag(7)-Ag(8)	2.9137(15)
Cu(5)-C(123)	1.901(13)	Ag(6)-Cu(6)	2.8196(19)	Ag(4)-P(4)	2.363(3)	Ag(8)-Ag(9)	3.0429(13)
Cu(5)-C(131)	1.892(13)	Ag(7)-Cu(4)	2.620(2)	Cu(1)-O(1)	2.043(8)	Ag(9)-Ag(10)	2.9496(13)
Cu(6)-C(139)	1.921(11)	Ag(7)-Cu(5)	3.0455(18)	Cu(4)-O(2)	2.031(9)	Cu(2)-Cu(3)	2.969(2)
Cu(6)-C(147)	1.923(13)						
P(1)-Ag(1)-Cu(1)	167.68(9)	P(2)-Ag(2)-Cu(3)	87.50(9)	P(3)-Ag(3)-Cu(5)	78.91(8)	P(4)-Ag(4)-C(68)	132.9(3)
P(1)-Ag(1)-Cu(2)	85.10(8)	P(2)-Ag(2)-Cu(4)	134.78(9)	P(3)-Ag(3)-Cu(4)	161.54(9)	P(4)-Ag(4)-Cu(6)	83.41(8)
P(1)-Ag(1)-Ag(10)	129.08(8)	P(2)-Ag(2)-Ag(7)	169.42(9)	P(3)-Ag(3)-Ag(8)	134.04(8)	P(4)-Ag(4)-Cu(1)	140.44(9)
P(1)-Ag(1)-Ag(5)	136.22(9)	P(2)-Ag(2)-Ag(8)	122.73(8)	P(3)-Ag(3)-Ag(7)	119.71(8)	P(4)-Ag(4)-Ag(5)	151.81(9)
C(59)-Cu(1)-C(67)	164.2(5)	C(139)-Cu(6)-C(147)	158.1(5)	C(99)-Cu(3)-C(91)	152.7(6)	C(131)-Cu(5)-C(123)	161.8(5)
C(59)-Cu(1)-Ag(1)	56.3(4)	C(139)-Cu(6)-N(4)	94.3(4)	C(99)-Cu(3)-N(2)	101.7(4)	C(131)-Cu(5)-N(3)	99.6(5)
C(59)-Cu(1)-Ag(4)	122.7(4)	C(147)-Cu(6)-N(4)	104.9(4)	C(91)-Cu(3)-N(2)	96.3(4)	C(123)-Cu(5)-N(3)	97.4(4)
C(59)-Cu(1)-Ag(5)	106.7(4)	C(139)-Cu(6)-Ag(4)	99.5(4)	C(99)-Cu(3)-Ag(2)	69.6(4)	C(131)-Cu(5)-Ag(6)	63.0(5)
C(59)-Cu(1)-Ag(10)	52.8(4)	C(147)-Cu(6)-Ag(4)	68.6(3)	C(91)-Cu(3)-Ag(2)	89.2(4)	C(123)-Cu(5)-Ag(6)	109.9(4)
C(67)-Cu(1)-Ag(1)	114.8(4)	N(4)-Cu(6)-Ag(4)	95.8(2)	N(2)-Cu(3)-Ag(2)	93.4(2)	N(3)-Cu(5)-Ag(6)	130.1(2)
C(67)-Cu(1)-Ag(4)	50.8(4)	C(139)-Cu(6)-Ag(6)	123.0(3)	C(99)-Cu(3)-Ag(8)	102.0(4)	C(131)-Cu(5)-Ag(3)	89.7(4)
C(67)-Cu(1)-Ag(5)	57.6(4)	C(147)-Cu(6)-Ag(6)	53.8(3)	C(91)-Cu(3)-Ag(8)	53.8(4)	C(123)-Cu(5)-Ag(3)	81.4(4)
C(67)-Cu(1)-Ag(10)	112.3(4)	N(4)-Cu(6)-Ag(6)	122.7(2)	N(2)-Cu(3)-Ag(8)	146.1(2)	N(3)-Cu(5)-Ag(3)	98.3(2)
C(59)-Cu(1)-O(1)	95.6(5)	C(139)-Cu(6)-Ag(9)	62.6(3)	C(99)-Cu(3)-Cu(2)	81.9(4)	C(131)-Cu(5)-Ag(9)	123.2(5)
C(67)-Cu(1)-O(1)	99.4(4)	C(147)-Cu(6)-Ag(9)	114.6(3)	C(91)-Cu(3)-Cu(2)	122.1(4)	C(123)-Cu(5)-Ag(9)	51.4(4)
O(1)-Cu(1)-Ag(1)	133.1(3)	N(4)-Cu(6)-Ag(9)	118.8(2)	N(2)-Cu(3)-Cu(2)	77.9(2)	N(3)-Cu(5)-Ag(9)	119.3(2)
O(1)-Cu(1)-Ag(4)	95.6(3)	C(139)-Cu(6)-Ag(10)	53.2(3)	C(99)-Cu(3)-Ag(6)	45.4(3)	C(131)-Cu(5)-Ag(8)	104.3(4)
O(1)-Cu(1)-Ag(5)	155.1(2)	C(147)-Cu(6)-Ag(10)	105.4(4)	C(91)-Cu(3)-Ag(6)	126.7(4)	C(123)-Cu(5)-Ag(8)	57.5(3)
O(1)-Cu(1)-Ag(10)	131.7(3)	N(4)-Cu(6)-Ag(10)	144.9(2)	N(2)-Cu(3)-Ag(6)	135.1(2)	N(3)-Cu(5)-Ag(8)	148.0(2)
C(75)-Cu(2)-C(83)	162.0(5)	C(107)-Cu(4)-C(115)	163.1(5)	C(99)-Cu(3)-Ag(9)	100.3(4)	C(131)-Cu(5)-Ag(7)	46.1(3)
C(75)-Cu(2)-N(1)	95.2(4)	C(107)-Cu(4)-Ag(2)	118.7(4)	C(91)-Cu(3)-Ag(9)	79.6(3)	C(123)-Cu(5)-Ag(7)	115.8(3)
C(83)-Cu(2)-N(1)	101.4(4)	C(107)-Cu(4)-Ag(7)	107.8(4)	N(2)-Cu(3)-Ag(9)	136.4(2)	N(3)-Cu(5)-Ag(7)	138.1(2)
C(75)-Cu(2)-Ag(1)	99.4(5)	C(115)-Cu(4)-Ag(2)	53.4(3)	C(3)-N(1)-C(2)	103.1(8)	C(32)-N(3)-C(31)	103.1(8)
C(83)-Cu(2)-Ag(1)	72.4(4)	C(107)-Cu(4)-Ag(3)	49.6(4)	C(3)-N(1)-C(1)	114.1(9)	C(32)-N(3)-C(30)	113.7(9)
N(1)-Cu(2)-Ag(1)	94.6(2)	C(107)-Cu(4)-Ag(8)	55.1(3)	C(2)-N(1)-C(1)	107.4(8)	C(31)-N(3)-C(30)	109.0(8)
C(75)-Cu(2)-Ag(10)	58.0(4)	C(115)-Cu(4)-Ag(3)	119.8(4)	C(3)-N(1)-Cu(2)	110.9(6)	C(32)-N(3)-Cu(5)	113.7(6)
C(83)-Cu(2)-Ag(10)	104.3(3)	C(115)-Cu(4)-Ag(7)	56.0(3)	C(2)-N(1)-Cu(2)	109.1(7)	C(31)-N(3)-Cu(5)	108.2(6)
N(1)-Cu(2)-Ag(10)	138.2(2)	C(115)-Cu(4)-Ag(8)	109.3(4)	C(1)-N(1)-Cu(2)	111.7(6)	C(30)-N(3)-Cu(5)	108.8(6)
C(75)-Cu(2)-Cu(3)	73.4(5)	C(107)-Cu(4)-O(2)	97.2(5)	C(2)-N(2)-C(5)	108.3(8)	C(31)-N(4)-C(34)	107.8(8)

C(83)-Cu(2)-Cu(3)	115.1(4)	C(115)-Cu(4)-O(2)	99.2(4)	C(2)-N(2)-C(4)	105.1(8)	C(31)-N(4)-C(33)	105.0(8)
N(1)-Cu(2)-Cu(3)	84.1(2)	O(2)-Cu(4)-Ag(2)	102.7(3)	C(5)-N(2)-C(4)	112.9(8)	C(34)-N(4)-C(33)	113.7(8)
C(75)-Cu(2)-Ag(5)	113.5(4)	O(2)-Cu(4)-Ag(3)	129.4(3)	C(2)-N(2)-Cu(3)	105.9(6)	C(31)-N(4)-Cu(6)	109.6(6)
C(83)-Cu(2)-Ag(5)	48.7(3)	O(2)-Cu(4)-Ag(7)	155.0(3)	C(5)-N(2)-Cu(3)	109.6(6)	C(34)-N(4)-Cu(6)	107.9(6)
N(1)-Cu(2)-Ag(5)	147.0(2)	O(2)-Cu(4)-Ag(8)	135.6(3)	C(4)-N(2)-Cu(3)	114.5(6)	C(33)-N(4)-Cu(6)	112.7(6)
